# Urinary Iodine Clearance following Iodinated Contrast Administration: A Comparison of Euthyroid and Postthyroidectomy Subjects

**DOI:** 10.1155/2014/580569

**Published:** 2014-11-12

**Authors:** Janice D. Ho, James F. Tsang, Kylie A. Scoggan, William D. Leslie

**Affiliations:** ^1^University of Manitoba, Winnipeg, MB, Canada R3T 2N2; ^2^Department of Radiology, University of Manitoba, Winnipeg, MB, Canada R3T 2N2; ^3^Sector Strategies Division, Risk Management Bureau, Safe Environments Directorate, Healthy Environments and Consumer Safety Branch, Health Canada, Ottawa, ON, Canada K1A 0K9; ^4^Department of Medicine (C5121), 409 Tache Avenue, Winnipeg, MB, Canada R2H 2A6

## Abstract

*Purpose.* To compare iodine clearance following iodinated contrast administration in thyroidectomised thyroid cancer patients and euthyroid individuals. *Methods.* A convenience population (6 thyroidectomised thyroid cancer patients and 7 euthyroid controls) was drawn from patients referred for iodinated contrast-enhanced computed tomography (CT) studies. Subjects had sequential urine samples collected up to 6 months (50 samples from the thyroidectomised and 63 samples from the euthyroid groups). *t*-tests and generalised estimating equations (GEE) were used to test for group differences in urinary iodine creatinine ratios. *Results.* Groups had similar urinary iodine creatinine ratios at baseline, with a large increase 2 weeks following iodinated contrast (*P* = 0.005). Both groups had a return of urinary iodine creatinine ratios to baseline by 4 weeks, with no significant group differences overall or at any time point. *Conclusions.* Thyroidectomised patients did not have a significantly different urinary iodine clearance than euthyroid individuals following administration of iodinated contrast. Both had a return of urinary iodine creatinine ratios to baseline within 4 weeks.

## 1. Introduction

Iodinated contrast is frequently given to enhance the diagnostic utility of computed tomography (CT). The contrast material results in a large increase in body iodine stores, which is progressively cleared over several weeks to months. A typical CT study uses about 100 cc of intravenous contrast material, which translates to about 30 g of iodine [1]. There is concern that this transient increase in iodine can compete with ^131^I and interfere with subsequent management, such as whole-body scans and treatment with radioactive iodine in thyroid cancer patients. In addition, many groups recommend iodine depletion diets to optimize the therapeutic response to radioactive iodine in these patients [2, 3]. Thus, avoidance of iodinated contrast material is particularly important as it can lessen the effectiveness of radioactive therapy [4, 5].

The duration of time required for body iodine stores to return to normal following iodinated contrast administration has not been well studied in thyroid cancer patients. Recommendations for avoidance of radioiodine administration after iodinated contrast vary from 4 weeks to 1 year, with most suggesting about 3 months [6]. The American Thyroid Association Guidelines for the Management of Differentiated Thyroid Carcinoma recommend that “iodinated contrast should be avoided if radioactive iodine therapy is planned within the subsequent few months” [3]. The European consensus guidelines also state that radioactive iodine administration should be postponed for two to three months after the event of iodine contamination [7]. These guidelines are largely based upon iodine clearance in euthyroid individuals [6]. The normal thyroid has a considerable storage pool of organified iodine [6] and since the body recirculates this iodine, thyroidectomised patients may possibly have a quicker elimination time than euthyroid individuals. If confirmed, then a briefer interval between iodinated contrast administration and subsequent radioiodine therapy could be considered and would expedite therapy.

The objective of this study is to compare iodine clearance following iodinated contrast administration in thyroidectomised thyroid cancer patients and euthyroid individuals.

## 2. Methods

### 2.1. Subjects and Setting

A convenience study population was drawn from patients referred to the Saint Boniface General Hospital Diagnostic Imaging Department for iodinated contrast-enhanced CT studies. Thyroidectomised thyroid cancer patients were notified of the study by members of the CancerCare Manitoba thyroid cancer disease site group who identified eligible patients requiring contrast-enhanced CT for clinical reasons. Eligible thyroidectomised patients had previously undergone total or near-total thyroidectomy for thyroid cancer and were on a stable dose of suppressive L-thyroxine therapy. Control euthyroid subjects were recruited from routine diagnostic CT referrals coded to receive iodinated contrast. Eligible control individuals included those without documented thyroid disease and who were not taking thyroid hormone replacement. Biochemical euthyroidism was established at the time of CT. The University Health Research Ethics Board approved the study for the University of Manitoba and signed and informed consent was obtained.

Exclusion criteria included pregnancy, other major sources of iodine including any history of amiodarone use ever or iodinated contrast in the preceding 12 months, treatment with lithium, abnormal renal function (serum creatinine > 170 umol/L), inability or unwillingness to participate, and control subjects with known thyroid disease or those who were found to have abnormal thyroid function or anticipated survival of less than one year.

Routine CT protocols used the same dye load (100 cc of Omnipaque 350, GE Healthcare) for all adult patients undergoing scanning of the neck, chest, abdomen, or pelvis. We recorded routine demographics and clinical characteristics including sex, age, height, weight, and any iodine-containing medications, vitamin supplements, or herbal products. Prior to the patient undergoing the iodinated contrast enhanced CT, a urine sample was collected for baseline assessment of urinary iodine. Subjects were asked to collect a urine specimen every 2 weeks for the first 8 weeks and then monthly up to 6 months for 9 collections in total. Menstruating women were asked to delay the timing of collection to avoid contamination.

### 2.2. Urinary Iodine Analysis

Urinary iodine concentration is currently the most practical biomarker for determining the iodine nutritional status [8, 9]. Approximately 90% of all iodine ingested in the diet will be excreted in the urine [10]. Urine samples were stored in a −60 °C freezer until analysis at the end of the study. All samples were batch-run for assessment of urinary iodine in conjunction with creatinine at Health Canada. Urinary iodine concentrations were determined using ammonium persulfate digestion followed by colorimetric analysis based on the Sandell-Kolthoff reaction according to a modified microplate method [11]. Urinary creatinine concentrations were determined by enzymatic IDMS-standardized two-point rate on an Ortho-Clinical Diagnostics Vitros 5, 1FS analyser. The urinary iodine to creatinine ratio (microg/g) was calculated as the measure of iodine clearance [12] as used by the third National Health and Nutrition Examination Survey (NHANES III) (1984–1994). A review concluded that spot urinary iodine concentration is a reliable measure of the iodine intake in the population when expressed as a function of urinary creatinine to correct for the influence of fluid intake.

### 2.3. Statistical Analysis

Demographic and clinical characteristics of the thyroidectomised thyroid cancer patients and of the euthyroid controls are described using medians, means, and standard deviations (SD) for continuous variables and frequencies and percentages for categorical variables. Group comparisons for continuous data were conducted with the Student's *t*-test for specific time points. Models based upon generalised estimating equations (GEE) were used to analyse combined data for all time points from 4 weeks onwards assuming an exchangeable correlation structure. Separate GEE models were then constructed to look for an effect of time since contrast administration or a time × group interaction. The dependent variable used for the primary analysis was the urinary iodine creatinine ratio. In a secondary analysis we also looked at change in urinary iodine creatinine ratio from baseline. A *P* value of 0.05 was considered significant. Statistical analyses were performed with SPSS Statistics software (Version 22).

## 3. Results

### 3.1. Study Population

We enrolled 6 thyroidectomised thyroid cancer patients and 7 control subjects. In total 50 samples from the thyroidectomised thyroid cancer group were compared to 63 samples from the control group. There were no missing collections from the control group; there were 4 missing collections from the thyroidectomised thyroid cancer group. The ages ranged from 34 to 87 years (mean age 58.3 years). Baseline demographics showed no significant difference between the two groups (Table 1).

The mean urinary iodine creatinine ratio was 248.2 microg/g at baseline for the thyroidectomised thyroid cancer patients and 138.3 microg/g at baseline for the control patients. As anticipated, the two-week mean values were still elevated above baseline (thyroidectomised thyroid cancer 770.3 microg/g versus 670.3 microg/g control groups).

**Figure 1:**
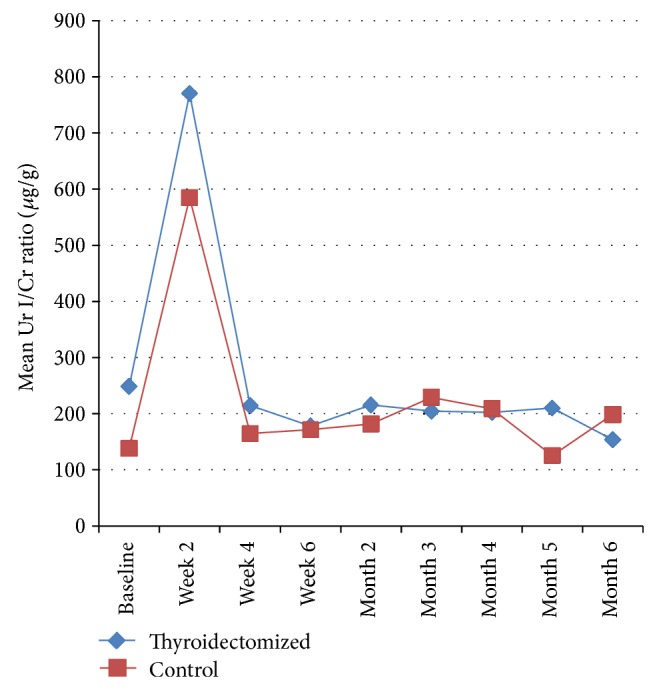
Mean urinary iodine creatinine ratios of thyroidectomised and control patients (microg/g).

Figure 1 shows the mean urinary iodine creatinine ratios for the two groups, demonstrating a return to baseline values 4 weeks following iodine contrast administration. Pairwise testing showed no significant group differences in urinary iodine creatinine ratios at baseline or any subsequent time point. When expressed as the change from baseline, only the 2-week sample showed significantly increased urinary iodine clearance (Table 2).

GEE was used to examine for differences in urinary iodine creatinine ratios between the two groups from week 4 onwards and no significant group effect was identified (*P* = 0.53). In a separate GEE model we did not identify any significant time effect (*P* = 0.19) or time × group interaction (*P* = 0.84). A GEE model with change from baseline from week 4 onwards showed a statistically significant difference between the two groups, lower in the thyroidectomised thyroid cancer group (parameter 113.1 microg/g [95% confidence interval −12.8 to −213.3 microg/g], *P* = 0.027).

## 4. Discussion

Following exposure to a large iodine load, the plasma concentrations of free iodine remain elevated as the body's iodine stores are expanded in the interstitial fluids and in the thyroid colloid. Current literature suggests that, in euthyroid individuals, the body's iodine stores are increased for at least 3 months following iodinated contrast [6]. Our study finds that the urinary iodine creatinine ratio returns to baseline by 4 weeks following iodinated contrast in both euthyroid and thyroidectomised thyroid cancer subjects. In addition, there was no significant difference between the thyroidectomised and euthyroid patients at 4 weeks, nor throughout the rest of the study period up to 6 months.

These results are similar to the previous report from Padovani et al. in which urinary iodine levels returned to baseline at one month in thyroidectomised thyroid cancer patients after receiving iodinated contrast [13]. A prospective study by Nimmons et al. showed that the median time for urinary iodine levels to normalize in euthyroid subjects was 43 days, with 75% of subjects returning to baseline by 60 days [14].

We acknowledge that our small study size was a limitation. Our study enrolled only 13 subjects in total and may have been underpowered to detect significant differences between the two groups. The small numbers of cases and controls are compensated for by the multiple time points assessed, which allowed for all urinary collections from 4 weeks onwards to contribute to the statistical analysis using GEE. In addition, thyroid cancer patients need to be on a low iodine diet prior to radioiodine treatment. While this study demonstrates that urinary iodine creatinine ratio returns to baseline by 4 weeks in both thyroidectomised thyroid cancer and euthyroid subjects, it does not look at the effect of a low iodine diet on the urinary iodine creatinine ratio post iodinated contrast. We did not collect data on noncontrast sources of exogenous iodine (e.g., diet, vitamin supplements), but our analysis of the change in iodine excretion from baseline partially compensates for interindividual differences in dietary iodine intake.

The best imaging modality to investigate thyroid cancer patients continues to be studied. Increasingly, CT is being compared to ultrasound, magnetic resonance imaging, or positron emission tomography techniques. As more information is gained about the sensitivity, specificity, and accuracy of these techniques, CT may be used less frequently which would ameliorate the concern of iodinated contrast and therapeutic radioactive iodine.

In conclusion, we found that 4 weeks may be sufficient for urinary iodine creatinine ratios to return to baseline value following iodinated contrast in both thyroidectomised thyroid cancer and euthyroid subjects. This suggests that clearance time may be more rapid than previously thought and that a shorter time interval between iodinated contrast and radioiodine therapy might be considered but requires confirmation in larger studies that include a broad case mix in terms of age and dietary iodine intake.

## Figures and Tables

**Table 1:** Group demographics of control and thyroidectomised individuals.

	Euthyroid control group	Thyroidectomised thyroid cancer patients
Sex	4 females, 3 males	4 females, 2 males
Age, years (SD)	58.6 (7.5)	61.0 (18.4)
Height, cm (SD)	168.0 (8.8)	165.8 (7.7)
Weight, kg (SD)	85.9 (19.3)	77.8 (17.4)
BMI, kg/m^2^ (SD)	30.4 (6.5)	28.0 (4.3)

**Table 2:** Urinary iodine creatinine ratio change from baseline (microg/g).

	All subjects microg/g (SD)	Thyroidectomised thyroid cancer group microg/g (SD)	Euthyroid control group microg/g (SD)
Week 2-baseline	481.3^*^ (511.8)	522.0 (562.9)	446.3 (506.6)
Week 4-baseline	−1.3 (111.9)	−33.5 (134.6)	26.3 (89.5)
Week 6-baseline	−7.5 (118.2)	−64.8 (120.1)	33.4 (106.3)
Month 2-baseline	8.8 (138.7)	−39.9 (102.4)	43.5 (157.8)
Month 3-baseline	28.7 (159.6)	−43.7 (142.0)	90.7 (156.7)
Month 4-baseline	17.0 (140.1)	−45.7 (88.0)	70.7 (159.7)
Month 5-baseline	−24.5 (79.1)	−38.2 (90.4)	−12.9 (73.3)
Month 6-baseline	30.7 (173.4)	−21.1 (25.1)	60.2 (216.8)

^*^ *P* = 0.005.
